# Micro- and macro-borderless surgery using a newly developed high-resolution (4K) three-dimensional video system

**DOI:** 10.1371/journal.pone.0250559

**Published:** 2021-05-12

**Authors:** Shintaro Yagi, Takashi Ito, Hisaya Shirai, Siyuan Yao, Yuki Masano, Eri Ogawa, Ryosuke Gabata, Shinji Uemoto, Eiji Kobayashi

**Affiliations:** 1 Department of Hepato-Biliary-Pancreatic Surgery and Transplantation, Kanazawa University, Kanazawa City, Ishikawa, Japan; 2 Department of HBP and Transplant Surgery, Kyoto University, Kyoto City, Kyoto, Japan; 3 Shiga University of Medical Science, Otsu City, Shiga, Japan; 4 Department of Organ Fabrication, Keio University School of Medicine, Tokyo, Japan; Imperial College Healthcare NHS Trust, UNITED KINGDOM

## Abstract

**Objective:**

Microsurgery using conventional optical microscopes or surgical loupes features a limited field of view and imposes a serious strain on surgeons especially during long surgeries. Here we advocate the micro- and macro-borderless surgery (MMBS) using a novel high-resolution (4K) three-dimensional (3D) video system. This study aimed to confirm the applicability of this concept in several surgical procedures.

**Methods:**

We evaluated the possible use and efficacy of MMBS in the following experiments in porcine subjects. Experiment 1 (non-inferiority test) consisted of dissection and anastomosis of carotid artery, portal vein, proper hepatic artery, and pancreatoduodenectomy with surgical loupe versus MMBS. Experiment 2 (feasibility test) consisted of intra-abdominal and intra-thoracic smaller arteries anastomosed by MMBS as a pre-clinical setting. Experiment 3 (challenge on new surgery) consisted of orthotopic liver transplantation of the graft from a donor after circulatory death maintained by machine perfusion. Circulation of the cardiac sheet with a vascular bed in experiment 2 and liver graft during preservation in experiment 3 was evaluated with indocyanine green fluorescence imaging equipped with this system.

**Results:**

Every procedure was completed by MMBS. The operator and assistants could share the same field of view in heads-up status. The focal depth was deep enough not to be disturbed by pulsing blood vessels or respiratory movement. The tissue circulation could be evaluated using fluorescence imaging of this system.

**Conclusions:**

MMBS using the novel system is applicable to various surgeries and valuable for both fine surgical procedures and high-level surgical education.

## Introduction

**Fig 1:**
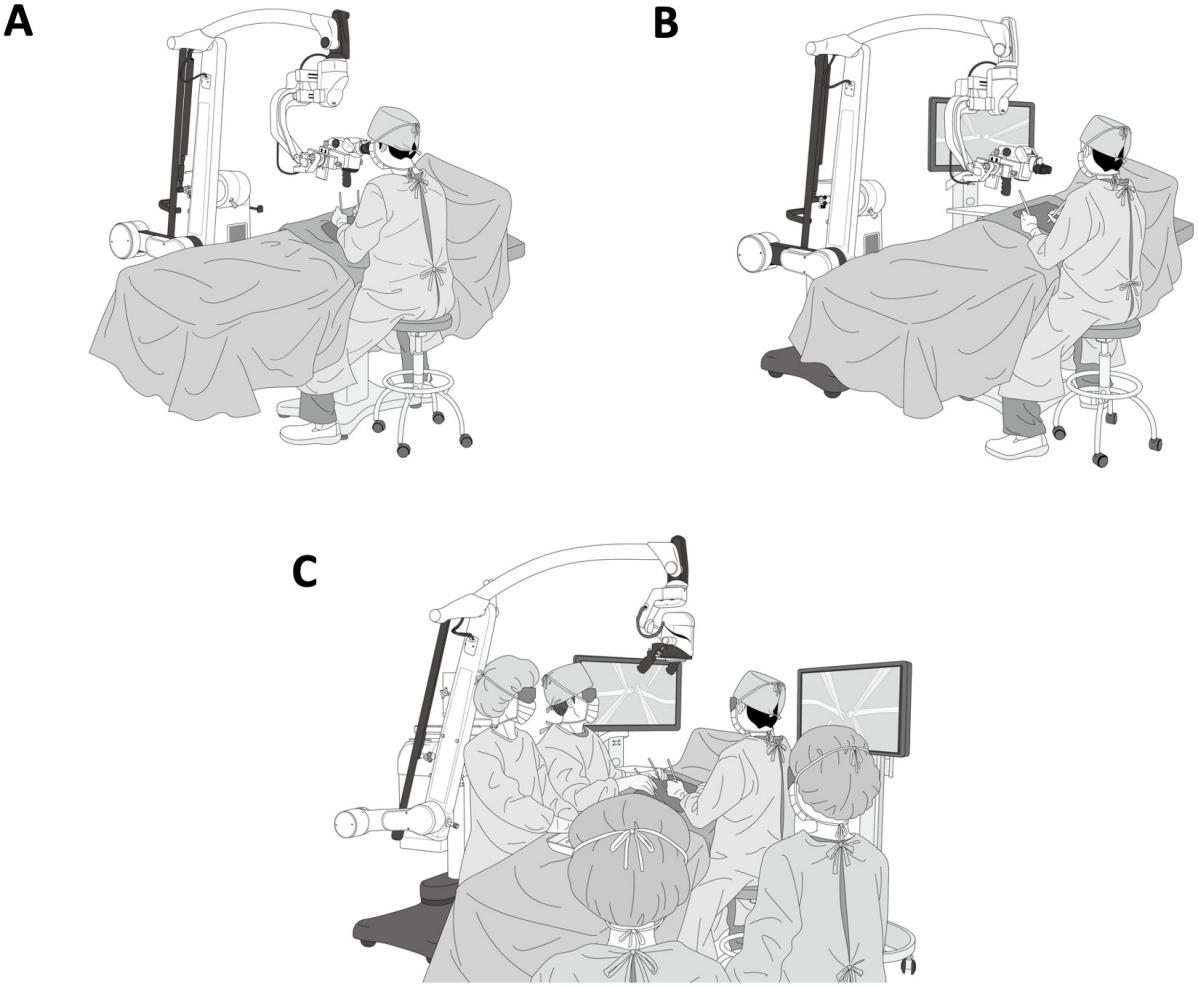
Past, present and future. (A) Past: microsurgery using conventional binocular optical microscopes. (B) Present: microsurgery using conventional optical microscopes with a monitor. (C) Future: micro- and macro-borderless surgery using novel high-resolution 4K- three-dimensional video system.

In 1989, laparoscopic cholecystectomy was introduced as an alternative to the open procedure. However, its significance was actually more than just technicality, it was a cultural revolution towards camera-assisted surgeries, whose fruits were thought to keep being harvested for decades [1]. And indeed, the use of laparoscopic and robot-assisted surgeries has been on the rise in gastrointestinal and thoracic surgery by the turn of the third millennium, enabling young surgeons to adapt to indirect surgeries with their “heads-up”. However, these indirect operations using laparoscopy or robots could not be applicable to microsurgery or fine vascular suturing because of a lack of fine surgical equipment as well as of haptic perception in the setting of the minimally invasive access. On the other hand, microsurgery using conventional binocular optical microscopes features a limited field of view and imposes a serious strain on surgeons especially during long surgeries (Fig 1A and 1B). Therefore, the heads-up approach was recently developed in neurosurgery [2] ophthalmological [3] surgery, and head and neck surgery [4], where the surgical field is displayed on a screen via a high-resolution camera just like in laparoscopic surgery [3].

With the introduction of the three-dimensional (3D) image into surgery, 3D tasks were believed to be more accurately and quickly performed than the ordinary two-dimensional ones [5]. This approach might be implemented in a greater variety of operations using a high-resolution 3D video camera as surgeons can perform a more efficient operation in a good posture watching 3D monitors and not forced to use an eyepiece thus reducing the overstrain of a long surgery. Using a conventional microscope system equipped with a monitor, the operator and assistants could not watch the monitors and operative field directly due to interference by the eyepiece of the microscope. For an optimum operative field, surgeons need stationary as well as seamless magnification with a single monitor featuring both high magnification such as that of the microscope and low magnification for visualization of the circumference of the operative field.

**Fig 2:**
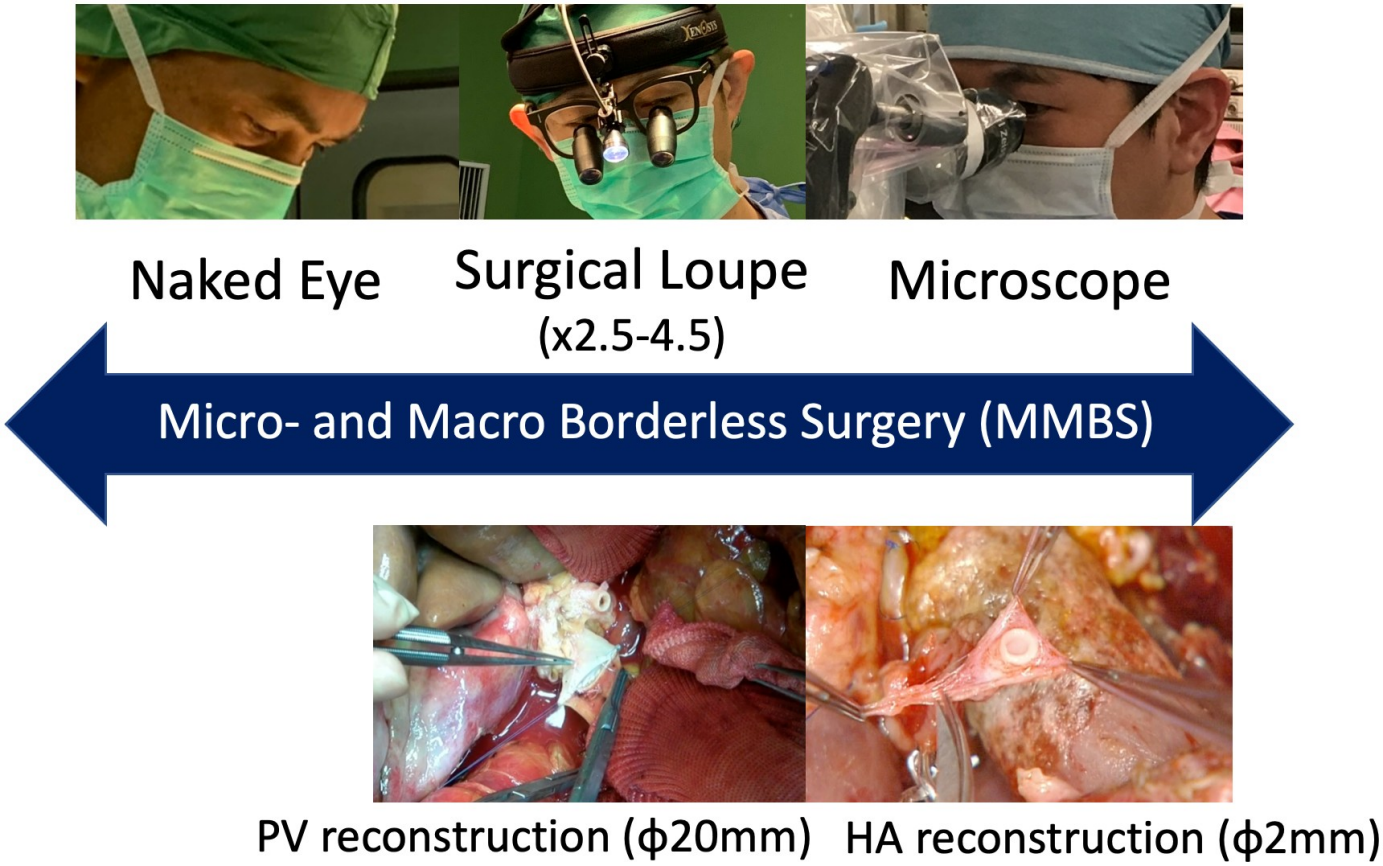
Application of “micro- and macro-borderless surgery” for various operations. PV, portal vein; HA, hepatic artery.

However, in some situations during microsurgery, especially in the abdomen and small arterial reconstruction, some obstacle such as: (A) Narrow field of view, (B) Deep operation field, (C) Respiratory movement that requires a significant depth of field (DOF) of the microscope, and (D) Setup of the microscope during the abdominal surgery takes approximately 15–30 minutes, which may consume more operative time. To overcome these issues, we developed the novel 4K-3D video system (Fig 1C) with a highly performing camera and fluorescence-equipped imaging technology advocating the micro- and macro-borderless surgery (MMBS) [6], which open a new window onto advanced microsurgery as well as “macro-surgery,” which is currently performed with the aid of a low-magnification surgical loupe (Fig 2). In this study, we evaluated the applicability of the MMBS using a newly developed high-resolution 4K-3D video system in various abdominal, thoracic, or other operations including tissue dissection and microsurgical anastomosis in large animals.

## Materials and methods

### MMBS concept

While the surgeon stands on one side of the operating table and the monitor is placed on the other side facing the operating table, the operator and the assistants perform the operation by watching the 3D monitor using an easy heads-up posture (Fig 1C). It is possible for the surgeons to quickly adjust the magnification that enables the surgeons to perform surgeries that require a high magnification to low magnification. We named this concept MMBS [6].

### Development of high-resolution 4K-3D video system

#### Seamless variable magnification

Zoomed-in and -out images can be displayed seamlessly on the monitors. Total magnification (TM) consists of objective magnification (OM) and monitor magnification (IM). TM is calculated by the following equation: TM = OM × IM, where IM is the diagonal length of the monitor/diagonal length of the imaging element. By selecting the monitor size, the TM could be up to 110 times (Table 1).

**Table 1:** The size of a 1-mm object on different displays according to display size.

WD	Display size	
(mm)	55-inch	31-inch
200	110	59
300	88	48
400	74	40
500	63	34
600	56	30
700	50	27
800	45	24
900	41	22
1000	37	20

WD, working distance.

### Rapid focus and magnification adjustment

This system has the ability to change the magnification from the lowest to highest in 1.8 seconds.

### High resolution and deep depth of field (DOF)

**Fig 3:**
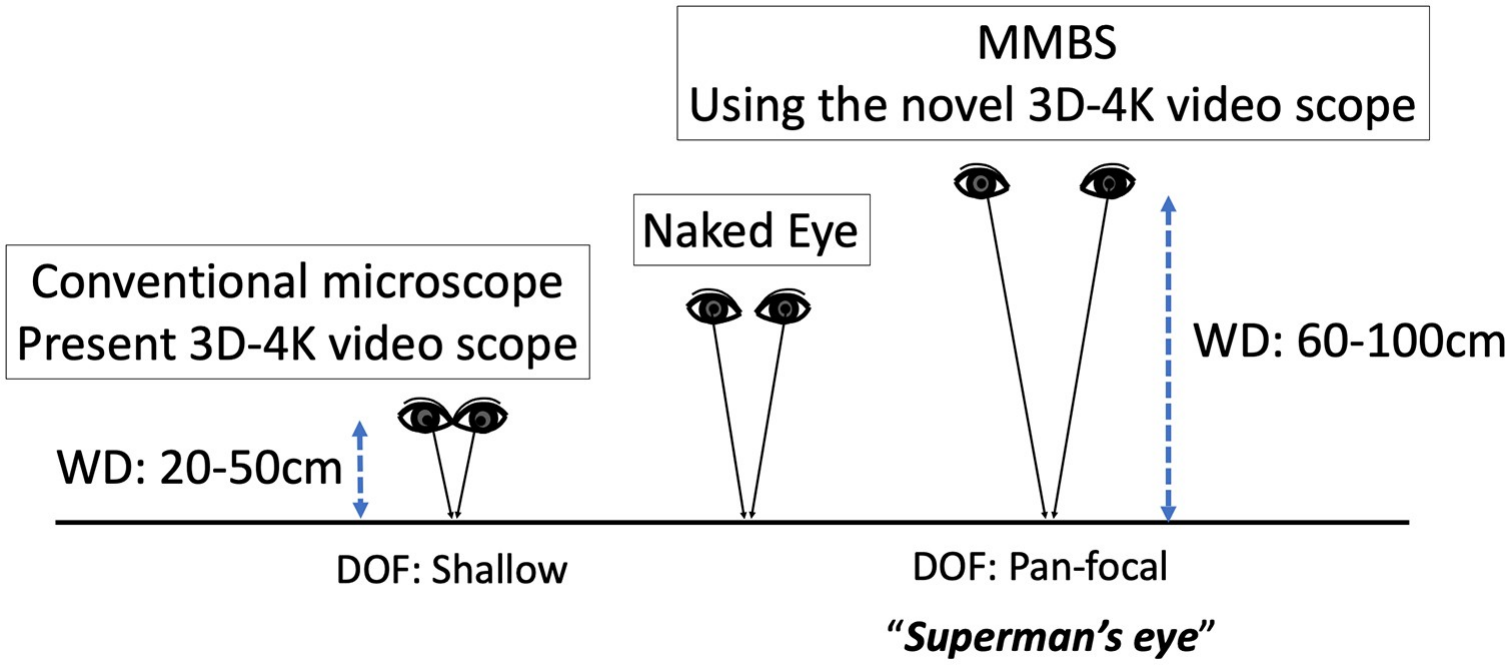
Achievement of binocular pan-focal zoon function in MMBS. The high-quality binocular pan-focal zoom function allows stereoscopic viewing of the naked eye and the microscope field of view without borders just like “Superman’s eye” due to the parallax difference. DOF, depth of field; MMBS, micro- and macro-borderless surgery; WD, working distance.

**Fig 4:**
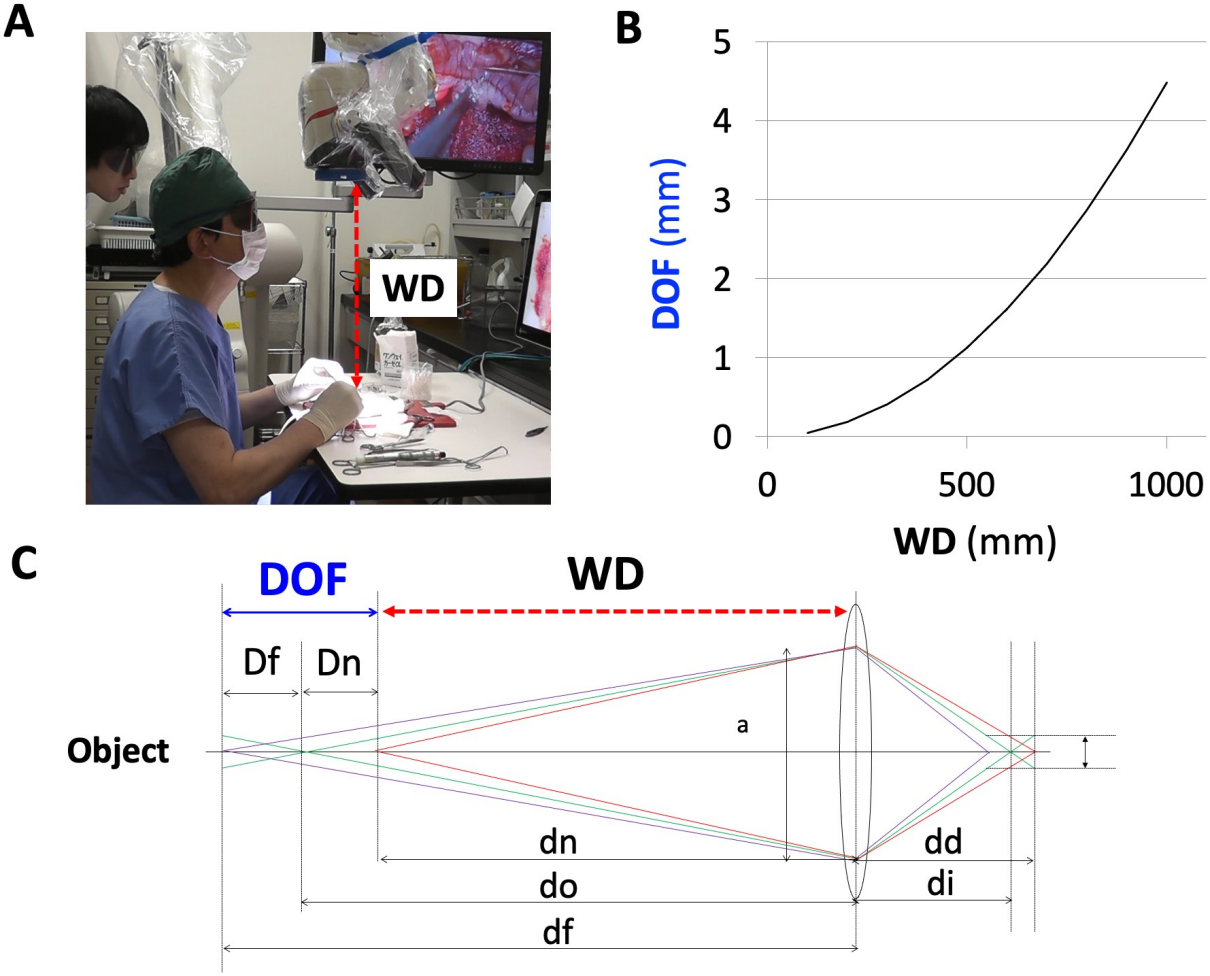
Relationship between DOF and WD. (A) WD (red line) in the operation. (B) Relationship between DOF and WD calculated with a lens F value of 5.6 and allowable circle of confusion of 4 μm. (C) Principal lens: DOF = Dn + Df. DOF correlates with WD (red line) (Please see Supplemental Digital Content S2 Fig). DOF, depth of field; WD, working distance.

**Fig 5:**
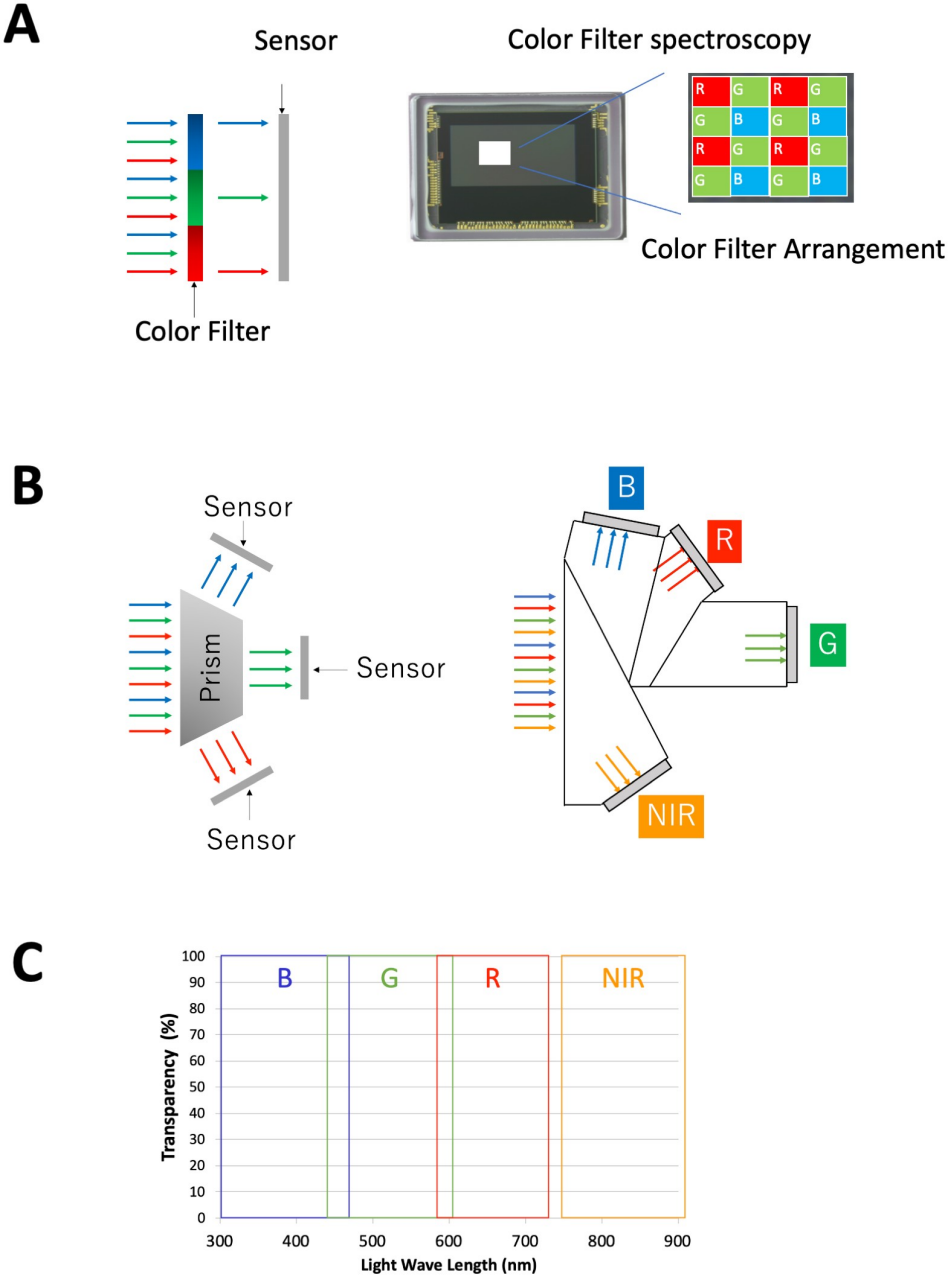
4-MOS of the 3D-4K video system. (A) Conventional single chip color sensor system. If it has only one sensor, the color filter absorbs most of the light beam. (B) Wavelength spectroscopic camera with prisms; Light passing through one lens is divided into four wavelengths: blue, green, red and near-infrared (NIR) by the prism. (C) Transparency and wavelength of the light to catch by the sensor; The subtly overlapping in the visible light region is to catch all the visible light. There is gap between visible light and NIR not to catch unnecessary NIR except ICG fluorescence. B, blue; R, red; G, green; NIR, near-infrared; MOS, metal oxide semiconductor.

This video system make it possible to achieve both high resolution (S1A Fig) and a deep DOF (S1B Fig). The DOF was about 4–5mm when the camera head was set above 100 cm from the operative field. This device achieved a high-quality binocular pan-focal zoom function that allows stereoscopic viewing without borders from the microscopic view to the naked eye view just like “Superman’s eye” due to the difference in parallax with a relatively large working distance of 60–100 cm (Fig 3). In the case of enlarging at a close distance using the microscope, since the triangle depicted by the light beam is flattened, much of the light reflected by the object can be captured by the lens and a bright, high-contrast, and clear image can be obtained; however, the DOF will be shallow. From the principle of the lens, DOF is proportional to working distance (Fig 4, S2 Fig). Using an objective lens from a distance, the triangle that the ray draws become a pointed shape, so the DOF increases (Fig 4C). However, since the reflected light of a distant object is largely enlarged, it is likely to be a dark, low-contrast, and unclear image. Therefore, we developed a video microscope combined with a high-quality optical lens and made the sensor of the camera a multi-plate 4-MOS (Fig 5) to enable the capture of a high-resolution 3D image. Accordingly, the disadvantage of a conventional microscope with a shallow DOF at the time of high magnification has been improved upon.

### Making sufficient working space and distance between the operative field and objective lens

Since the monitor is placed opposite the surgeon on the operating table, the objective lens is placed at a higher position above 100 cm from operating table, enabling both the operator and the assistants to visualize the operative field directly without disturbance by the objective lens (S1 Video).

### Projection of invisible light imaging

The camera has two imaging elements of visible and invisible light, which can both be projected onto the monitor and used in conjunction with fluorescence imaging, using indocyanine green (ICG), to confirm the blood flow (S2 Video).

### Color and near-infrared light

This 4-Metal Oxide Semiconductor (MOS) microscope and camera system is the world’s first to enable simultaneous shooting of visible and near-infrared (NIR) light. For high sensitivity, three visible lights (red, green, and blue) and ICG fluorescence wavelength light are separated by four prisms and captured by four independent sensors. Each light reaching the sensor is individually amplified by a parallel signal processing circuit, so a clear visible light image and a low-noise NIR image can be superimposed (Fig 5B and 5C). A high-speed signal processing circuit realizes a delay of less than 1/60 seconds.

### Large animal experiments to evaluate MMBS ability using a novel 4K-3D video system

All animal experiments were approved by the animal research committee: Experiment 1 by the Kobe Medical Devise Developing Center (IVT17-24), Experiment 2 by Fukushima Medical Devise Developing Support Center (20191204–1), and Experiment 3 by the Kobe Medical Devise Developing Center (IVT19-01).

### Animals

All animal experiments complied with the ARRIVE guidelines and were carried out in accordance with the National Institutes of Health Guide for the Care and Use of Laboratory Animals (NIH Publications No. 8023, revised 1978). These experiments were conducted according to non-Good Laboratory Practices and a reliability criterion. Two female porcine subjects in Experiment 1 and one female porcine subject in Experiment 2 and 3 weighing 25 kg were used. All animals were sacrificed after the completion of all procedures by the administration of KCL under high-concentration (5%) isoflurane inhalation.

### Design of animal experiments

We evaluated the possible use and efficacy of MMBS in the following 3 experiments. All of the operative procedures except for the surgical loupe group in Experiment 1 were performed by seeing the monitor set in front of the operative table using the high-resolution 4K-3D video system (MMBS concept).

#### Experiment 1: Non-inferiority test

Portal vein, hepatic arterial reconstruction, and pancreaticoduodenectomy were evaluated in porcine subjects using MMBS or surgical loupe. Two porcine subjects were used as liver transplant and pancreaticoduodenectomy models and operated on by expert hepatobiliary pancreas and transplant surgeons. The operations were performed using only a surgical loupe (SuperVu Prismatic system 2.5x and 4.5x, Keeler Instrument Inc, PA, USA) with a magnification of 2.5–4.5× in one subject and using MMBS in another porcine subject.

#### Experiment 2: Feasibility test

For further evaluation of the applicability for smaller arterial anastomosis in the abdominal and cardiovascular surgery, we performed a left hepatic arterial anastomosis and anastomosis between the internal thoracic artery and a cardiac sheet with vascular bed derived from porcine small intestine [7]. The circulation of the transplanted cardiac sheet was confirmed using ICG fluorescence equipped with this novel system.

#### Experiment 3: Challenge on a recently developed ischemia-free liver transplantation

In order to address the challenges of a new surgery, we performed ischemia-free liver transplantation [8] of the liver graft from a donor after circulatory death (DCD) in the porcine model. The tissue circulation of the liver graft was confirmed by ICG fluorescence during the preservation period.

### Anesthesia and operative procedures

After 12 h of fasting, anesthesia was induced by the intramuscular administration of ketamine hydrochloride (5 mg/kg) and atropine sulphate (1 mg/body) followed by endotracheal intubation and maintenance with oxygen, nitrous oxide, and isoflurane by positive pressure mechanical ventilation.

#### Experiment 1

These following procedures were performed using only a surgical loupe in 1 porcine and was performed using only a 4K-3D video system in another porcine: (1) hepatic arterial anastomosis (3.5 mm in diameter) with interrupted 9–0 polypropylene sutures (9–0 ASFLEX, Crown Jun Kohno, Tokyo, Japan); (2) portal vein (PV) anastomosis (10 mm in diameter) with continuous 6–0 ASFLEX; (3) dissection of the pancreas; (4) pancreaticojejunostomy; anastomosis between main pancreatic duct (1.5 mm in diameter) and jejunum with an internal stent using a 24G catheter by interrupted 6–0 ASFLEX with modified Blumgart anastomosis [9] between pancreas and serosa of the jejunum using 4–0 polypropylene sutures (OIIN, Crown Jun Kohno).

The surgical procedures including dissection of the vessels and anastomosis were compared in terms of feasibility and the posture of the surgeons and assistants.

#### Experiment 2

One porcine subject was subjected to the following: (1) exposure and anastomosis of left hepatic artery (1.5 mm in diameter) with interrupted 9–0 ASFLEX; (2) exposure of the internal thoracic artery after a sternal midline incision; (3) anastomosis between the internal thoracic artery and veins (3 mm in diameter) and the cardiac sheet derived from the porcine intestine using the tissue engineering technique with 9–0 ASFLEX; (4) confirmation of the blood flow of the cardiac sheets using ICG fluorescence imaging after injection of 2.5 mg/body ICG (Diagnogreen, Daiichi-Sankyo, Tokyo, Japan) intravenously.

#### Experiment 3

Ischemia-free liver transplantation was demonstrated with an orthotopic method of another study [8]. Briefly, in the donor operation, an uncontrolled DCD model was established by blockade of circulation through clamping of the thoracic aorta and setting of the warm ischemic time to 120 minutes; during procurement of the liver graft, catheters were inserted into the hepatic artery and PV to allow continuous perfusion of the graft by the machine perfusion system until immediately before reperfusion in the recipient. In the recipient operation, first, the vein grafts from the donor were anastomosed side-to-end to infrahepatic inferior vena cava (IHIVC) with 5–0 polypropylene sutures (5–0 PROLENE, Ethicon, Somerville, NJ, USA), and an active shunt circuit was established from the splenic vein and IHIVC graft to the left jugular vein. After the recipient’s liver was removed, anastomosis of suprahepatic inferior vena cava (SHIVC) of the liver graft and recipient was performed with 5–0 PROLENE under continuous perfusion of the recipient and implanted liver graft. Subsequently, PV with 6–0 PROLENE, and IHIVC with 5–0 PROLENE were anastomosed using intubated anastomosis technique [10]. During the preservation period, 1 mg/L of ICG (Diagnogreen) was injected into the perfusion solution to evaluate tissue circulation at the level of the liver graft.

## Results

### Experiment 1: Non-inferiority test

**Fig 6:**
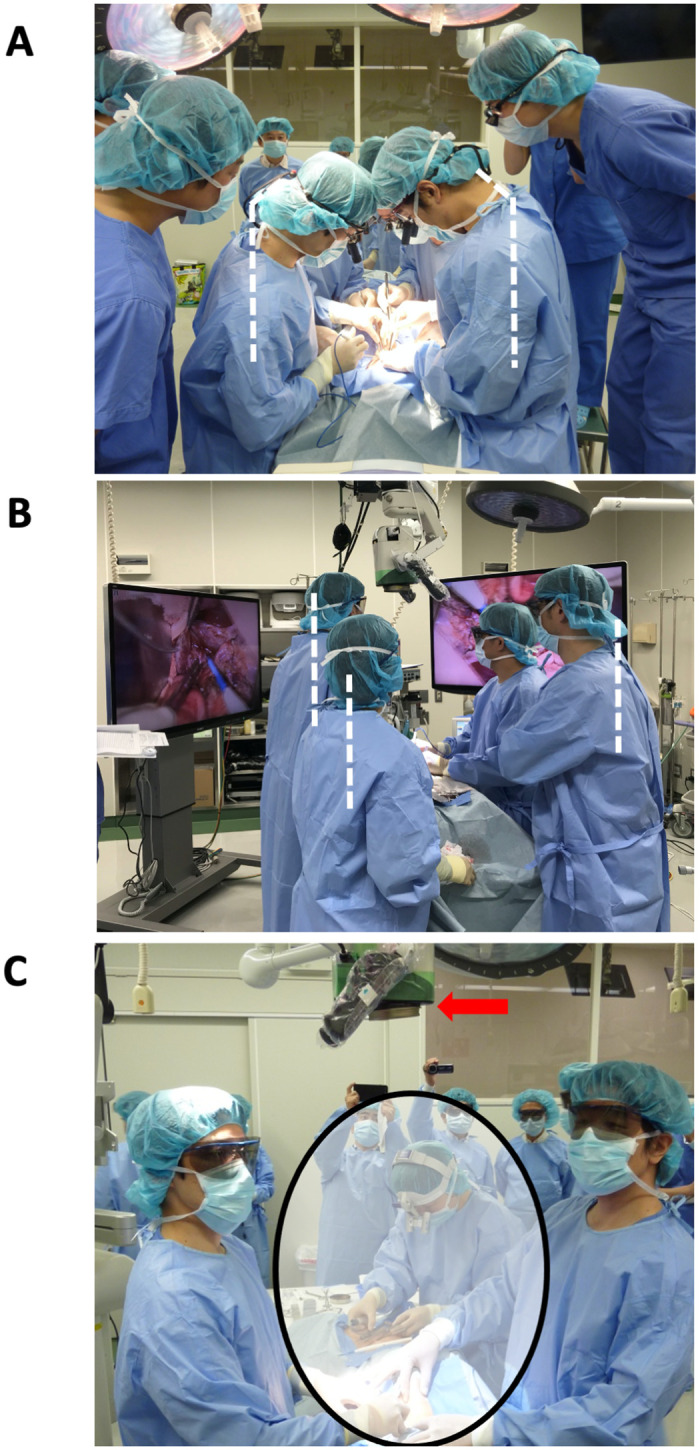
Posture of surgeons during operation. In conventional surgery using a surgical loupe, it was necessary to bend the neck nearly 60 degrees to visualize the surgical field (A). However, the MMBS made it possible for the surgeon to assume a comfortable position without bending the neck (B). Because the camera head (red arrow) is over the visual field, the surgeons can watch the monitors with sufficient space over the operative field (C). MMBS, micro- and macro-borderless surgery.

It takes approximately 10 minutes to set up the devise (5 minutes for connection and start-up + 5 minutes for draping, for a total of 10 minutes). The devise does not require any special start-up running operation. In the both surgical loupe and MMBS using 4K-3D video system groups, dissection and anastomosis in the PV, and common hepatic artery were completed without complications as well as pancreaticoduodenectomy (MMBS procedures shown in S3 Video). The procedural time were 22min vs. 30min for hepatic artery anastomosis (Surgical loupe vs. MMBS), 20min vs. 20min for PV anastomosis, 15min vs. 11min for dissection of the pancreas, 41min vs. 33min for pancreaticojejunostomy, that were comparable between surgical loupe and MMBS groups. In conventional laparotomy using a surgical loupe, it was necessary to visualize the surgical field by bending the neck nearly 60 degrees for a long period of time (Fig 6A). However, the new heads-up surgery using MMBS has made it possible to perform surgery in a comfortable position without the need to bend the neck (Fig 6B) with enough working space (Fig 6C). In the MMBS group, the operator, assistant, and observers could share the same operative field and maintain a heads-up status during surgery, which was not possible in the surgical loupe group (Fig 6B).

### Experiment 2: Feasibility test

The left hepatic artery with a 1.5-mm diameter in a porcine model could be completed by MMBS using the 4K-3D video system (S4 Video). The internal thoracic artery and vein and cardiac coronary artery were prepared using MMBS. Anastomoses of the artery and vein of the cardiac sheet to the internal thoracic artery and vein were also achieved by MMBS, followed by confirmation of the circulation using ICG fluorescence imaging equipped with the 4K-3D video system (S5 Video).

### Experiment 3: Challenge on a new surgery of ischemia-free liver transplantation

MMBS was conducted using the following procedures: (1) liver-graft procurement, (2) catheter insertion at the PV and hepatic artery of the procured liver graft, (3) anastomosis of the vein graft to the recipient’s PV and IHIVC, and (4) SHIVC anastomosis and PV anastomosis between the graft liver and recipient (S6 Video). Tissue circulation at the level of the liver graft was confirmed on the overlay images of ICG fluorescence (S3 Fig) during the preservation period.

## Discussion

We developed a novel high-resolution 3D video system equipped with fluorescence imaging that can rapidly magnify and confirm the blood flow in the vessels as well as organ perfusion using the new MMBS concept with the heads-up status of the surgeons, which could be applied in various surgical fields. Although the heads-up posture in microsurgery has been reported in ophthalmology, neurosurgery and head and neck surgery [2–4], the system is still under development throughout the world [11–14]. An 8K microscope is also under development [15, 16], however no 8K-3D video microscope has been developed to date.

While conventional video microscope systems have a working distance of 200 to 600mm, the new system has a working distance of 200 to 1000mm, making it possible to flexibly respond to a variety of procedures. In addition, the new system is equipped with a newly designed optical system with an 8x zoom that enables the resolution of a 4K camera, allowing observation from low to high magnification. The on-board 4K camera sensor is a new 4-chip type that can simultaneously perform white light observation and near-infrared fluorescence observation, making it possible to clearly observe ICG blood flow in real time. In brief, our system differs greatly from previous 4K-3D video systems in term of its large working space due to the high-performance objective camera with 4- MOS. Belykh E. et al. [17] reported the feasibility of microvascular anastomosis under 3D video microscopy in neurosurgery; however, at the highest magnification, the depth perception was inferior to that provided by a standard operative microscope, which impedes the procedure. Therefore, here we developed a novel 4K-3D video system with high magnification from a distance of 100 cm and a deep focus just like “Superman’s eye” (Fig 3).

This device is equipped with excitation of the ICG fluorescence of the blood flow and liver tumors such as hepatocellular carcinoma or metastatic liver tumors superimposed on a 3D monitor in real time, which would be applicable to navigation surgery [18], such as liver, lung, or sentinel lymph node resection. Surgical quality and safety will increase when blood flow and tumor location are confirmed using this device.

Epstein et al. [19] reported that the morbidity rate of work-related musculoskeletal disorders among surgeons is quite high; the rate for cervical disorders is 17%, higher than that of 1997, and 12% surgeons retire due to musculoskeletal disorders. When the cervical spine is bent to 60 degrees, the normal 5-kg force on the neck becomes 27 kg [20]. This MMBS was developed to enable surgeons to perform the operation with a heads-up position to view the monitor with seamless various magnification levels and provide the assistants with the same operative fields as the surgeon’s (Fig 6). By using this device instead of the surgical loupe, which forces the surgeon to tilt the neck to see the surgical field, it is possible to reduce the surgeon’s physical burden during surgery which would help reduce the incidence of work-related musculoskeletal disorders of the neck, it would also facilitate the educating the young surgeons about fine surgical techniques via allowing them to share the same surgical view and record and monitor their performance using the video system.

MMBS should facilitate microsurgical vascular anastomosis. In order to optimize the new system, it is necessary for the surgeon and assistants to be well trained in order to become familiar with the device. Furthermore, the position of the monitors needs to be carefully considered so that both the surgeon and the assistant can see the monitors well depending on the area to be operated on. Our next goal is to create a database of surgical techniques that can be used to train future surgeons by taking advantage of this new system. In addition, we can use this system to capture the digital data of the projected surgical field. Since the digital data can be streamed live, it may be possible to connect with an authoritative surgeon in a distant location as an advisor for a difficult operation.

Recently, organ fabrication in the field of regenerative medicine has advanced rapidly. However, its clinical application requires performing fine surgical techniques using a microscope or surgical loupes, which is challenging to surgeons, particularly those involved in basic research. To overcome these hurdles, the newly developed 4K-3D video microscope allows microsurgery in an easy heads-up posture by the operator and can be applied in highly complex surgical procedures of vascular reconstruction and regenerative medicine.

This newly developed 4K-3D video system may also improve the safety of the surgery itself, as everyone observing have the same field, and they can simultaneously check the exact tumor location and assure the blood flow. However, there are some limitations of this MMBS concept. It would be difficult to perform procedures with MMBS in areas where light cannot penetrate from above, such as the posterior and lateral walls, as with conventional microscopes. In the next step, we will evaluate this concept using our developing 4K-3D video system in a clinical study.

In conclusion, this novel MMBS concept using a 4K-3D video system with a heads-up posture is valuable for micro- and macro-borderless fine surgical procedures in various operations as it aids less straining surgeon posture and can facilitate high-level surgical education.

## Supporting information

S1 Fig Achievement of a (A) high resolution and (B) deep depth of field. (A) The 9–0 and 12–0 polypropylene threads and needles are clearly seen. (B) Depth of field was 4–5 mm when the working distance was 100 cm.
(TIFF)

S2 Fig (A) Principles of a lens. (B) Formula of depth of field (DOF). (C) Relationship between DOF and working distance. (TIFF)

S3 Fig Evaluation of tissue circulation of the liver graft during the preservation period. Indocyanine green (ICG) fluorescence imaging of the graft during the preservation period. (A) Monitor image display without ICG and (B) in near-infrared ICG mode; the green part represents ICG in circulation.
(TIFF)

S1 Video Micro- and macro-borderless surgery using a newly developed high-resolution 4K-3D video system in porcine subject. (MP4)

S2 Video Fluorescence imaging using indocyanine green to confirm the blood flow after orthotopic liver transplantation in a rat subject. (MP4)

S3 Video Micro- and macro-borderless surgery in dissection and anastomosis in the Portal Vein (PV), common hepatic artery and pancreaticoduodenectomy in a porcine model. (MP4)

S4 Video Micro- and macro-borderless surgery in anastomosis of the left hepatic artery in a porcine model. (MP4)

S5 Video Anastomoses of the artery and vein of the cardiac sheet to the internal thoracic artery and vein by micro- and macro-borderless surgery, followed by confirmation of the circulation using indocyanine green fluorescence imaging in porcine subject. (MP4)

S6 Video Ischemia free liver transplantation by MMBS in porcine model. (MP4)
