# Supplementary figures and images for: Micro- and macro-borderless surgery using a newly developed high-resolution (4K) three-dimensional video system
Source: PLoS One. 2021 May 12;16(5):e0250559. doi: 10.1371/journal.pone.0250559 (PMC8115828; doi:10.1371/journal.pone.0250559)

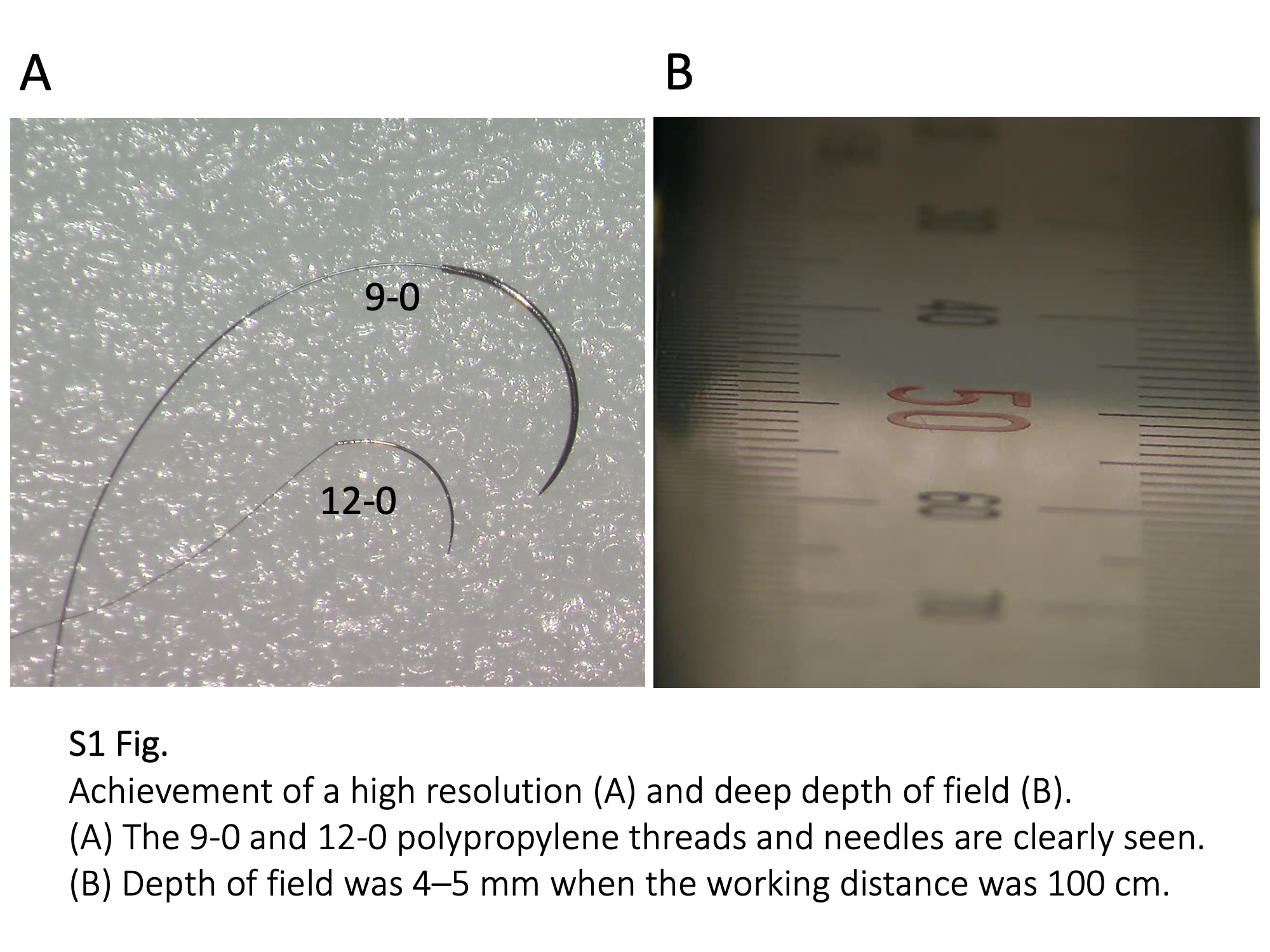

Supplement: S1 Fig — (A) The 9–0 and 12–0 polypropylene threads and needles are clearly seen. (B) Depth of field was 4–5 mm when the working distance was 100 cm. (TIFF) [file pone.0250559.s001.tiff]

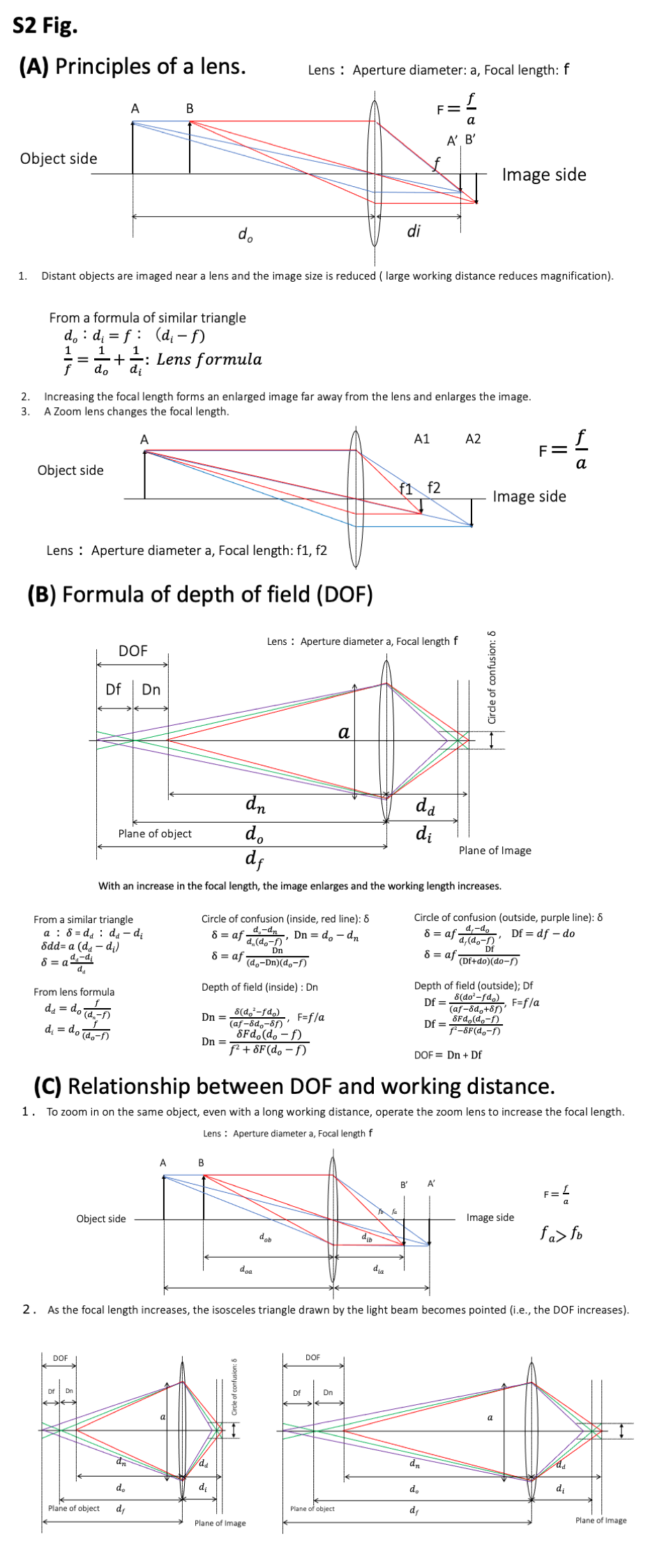

Supplement: S2 Fig — (TIFF) [file pone.0250559.s002.tiff]

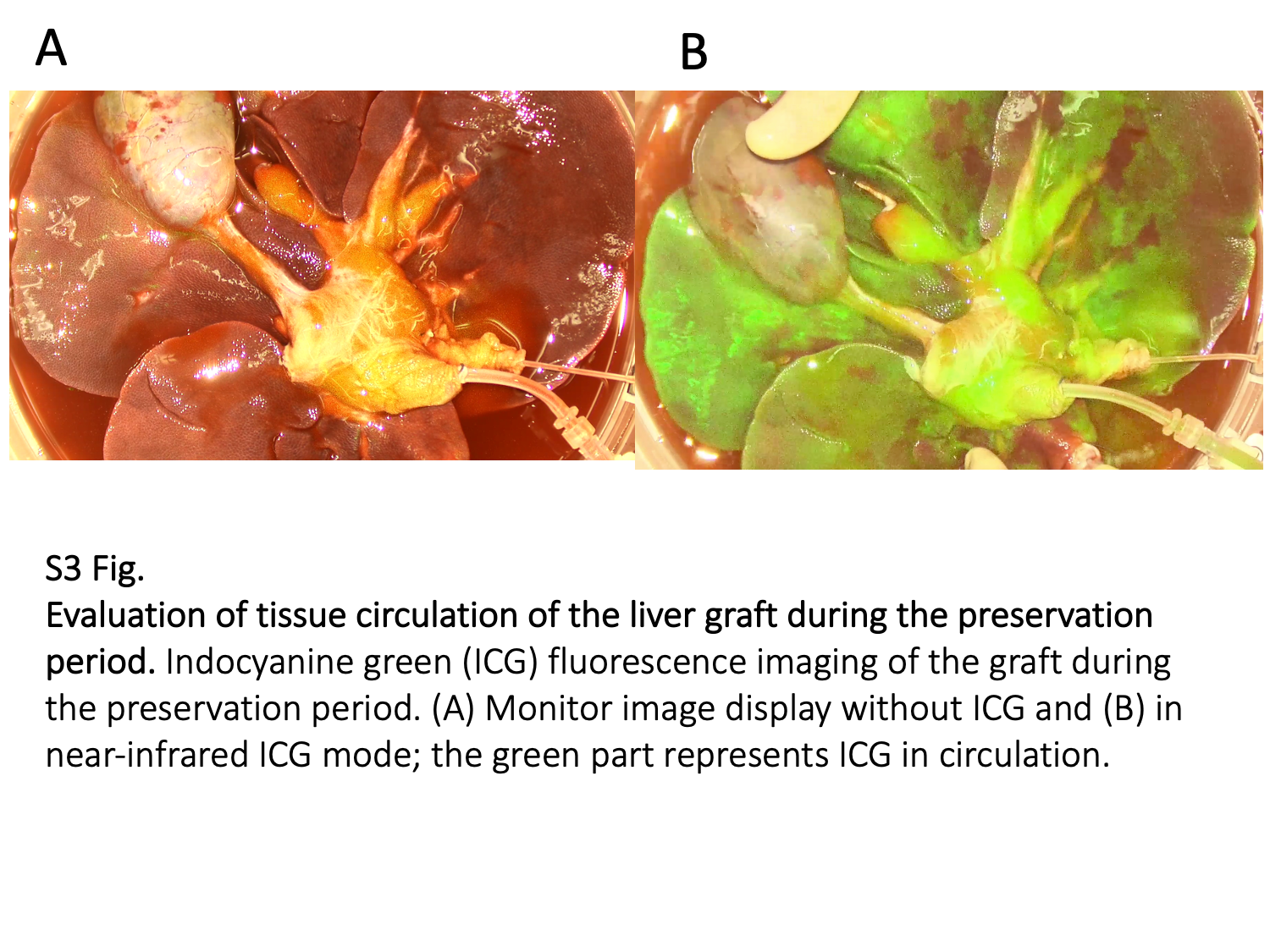

Supplement: S3 Fig — Indocyanine green (ICG) fluorescence imaging of the graft during the preservation period. (A) Monitor image display without ICG and (B) in near-infrared ICG mode; the green part represents ICG in circulation. (TIFF) [file pone.0250559.s003.tiff]
